# Effect of a Support on the Properties of Zinc Oxide Based Sorbents

**DOI:** 10.3390/nano12010089

**Published:** 2021-12-29

**Authors:** Maciej Chomiak, Bartłomiej M. Szyja, Marta Jędrysiak, Janusz Trawczyński

**Affiliations:** Department of Fuels Chemistry and Technology, Faculty of Chemistry, Wrocław University of Science and Technology, Gdańska 7/9, 50-344 Wrocław, Poland; maciej.chomiak@pwr.edu.pl (M.C.); 225916@student.pwr.edu.pl (M.J.)

**Keywords:** hot coal gas, desulphurization, sorption capacity, phase transition

## Abstract

We present the comparative analysis of three Zn-based sorbents for the process of sulphur removal from hot coal gas. The sorbents were prepared by a slurry impregnation of TiO_2_, SiO_2_ and Al_2_O_3_, resulting in complex, multiphase materials, with the dominant phases of Zn_2_TiO_4_, Zn_2_SiO_4_ and ZnAl_2_O_4_, respectively. We have analyzed the effect of supports on the phase composition, texture, reducibility and H_2_S sorption. We have found that the phase composition significantly influences the susceptibility of the investigated materials to reduction by hydrogen. Zn_2_TiO_4_ have been found to be the easiest to reduce which correlates with its ability to adsorb the largest amount of hydrogen sulphide—up to 4.2 gS/100 g—compared to the other sorbents, which absorb up to 2.2 gS/100 g. In the case of Zn_2_SiO_4_ and ZnAl_2_O_4_, this effect also correlates with reducibility—these sorbents have been found to be highly resistant to reduction by hydrogen and to absorb much less hydrogen sulphide. In addition, the capacity of ZnAl_2_O_4_ for H_2_S adsorption decreases in the subsequent work cycles—from 2.2 gS/100 g in the first cycle to 0.8 gS/100 g in the third one. Computational analysis on the DFT level has shown that these materials show different thermodynamic stability of sulphur sites within the unit cells of the sorbents. For Zn_2_TiO_4_ and Zn_2_SiO_4_, the adsorption is favorable in both the first and second layers of the former and only the top layer of the latter, while for zinc aluminate it is not favorable, which is consistent with the experimental findings.

## 1. Introduction

The large increase in energy consumption in the world enforces searching for new, more efficient and clean energy technologies. Supercritical fluidized-bed boilers and polygeneration technologies based on coal gasification seem to be very promising technologies for the efficient production of cheap and clean energy from coal [1,2,3,4]. Integrated Gasification Combined Cycle (IGCC) and Integrated Coal Gasification Fuel-cell Combined cycle (IGFC) technologies have already reached higher efficiency than conventional steam-powered plants; however, the upper limits of their efficiency have not been achieved yet [2]. One of the issues is the removal of the H_2_S from the hot coal gas, which is needed due to highly corrosive properties of this compound [5]. The practical realization of H_2_S removal at high temperature can improve the efficiency of the entire IGCC plant even further, and the development of viable, regenerable sorbents of hydrogen sulfide is highly demanded.

Practical realization of hot coal gas desulphurization still needs to overcome the problems associated with the gas composition and the process conditions. First of all, the sorbents are exposed to the reactions of reduction and carburization due to the presence of hydrogen, carbon monoxide and methane in the reaction environment. Moreover, carbon deposits can accumulate on the surface of the sorbents. These phenomena are undesired as they can lead to a decrease in the sorptive properties [6]. Sorbents for hot coal gas desulphurization should be characterized by high sorption capacity and the ability to remove H_2_S from the range of few ppm to several thousand ppm [7]. Moreover, the regeneration of the sorbents should be simple and should not lead to the formation of sulfates.

Pure ZnO based sorbents are perhaps the most efficient H_2_S sorbents due to their favorable sulphidation thermodynamics, high affinity to H_2_S [8,9] as well as price and availability [7,9]. These materials, however, are of limited use in high temperature H_2_S removal from hot gas due to the significant issues related to the regeneration [5]. Therefore, mixed oxides of Zn with Fe, Cu, Mn, Ce, Co, V and Ca have been proposed to have improved properties [6,10]. These materials can be used in two forms: (i) sorbents in which the active components are dispersed on the inert support or (ii) bulk mixed oxide often of spinel or perovskite structures [5].

As far as supported sorbents are concerned, an interesting approach is to utilize the mesoporous silica materials such as MCM-41 to support the Zn-based sorbents [11,12,13]. The formation of the mixed oxide—ZnSiO_4_—has been observed, with little influence on the performance of the material, however.

Carbon materials [14,15,16], natural aluminosilicates [17,18] semi-coke [19,20], nonmetallic oxides [21,22,23] and other materials [24] were studied as the complex, multicomponent H_2_S sorbents. Among impregnated sorbents, active phases often consist of simple metal oxides [21,22]. These systems, however, are relatively easily reduced to metallic form, usually less active in H_2_S sorption [10].

The advantage of bulk type sorbents is the higher content of active ingredients and, consequently, the greater sorption capacity, with the assumption of sulphur ions diffusion to the bulk [25]. Additionally, Lew et al. have proven that mixed metal oxides (Zn-Ti-O) exhibit better properties than a simple oxide ZnO [26] in terms of the rate of zinc loss and, as a consequence, stability in cyclic work. Many of these materials are also interesting as far as applications other than H_2_S capture are concerned, especially when they can serve as the H_2_S sensors [27,28].

As far as the desired characteristics of the sorbents are concerned, it was found that specific surface area of the sorbent has a significant impact on its activity [21]. However, at higher temperature range, this factor is less important, and it seems that focus has to be on ensuring a high theoretical sorption capacity to meet the level of the bulk sorbents. For both types of systems, the key issue is preventing the negative effects of metal oxide reduction, zinc migration on surface, zinc evaporation, metals sulfate formation, carbon deposition, etc. Another important criterion for sorbent selection is also the possibility of its simple regeneration between sorption cycles [5,29,30,31,32,33].

In the case of zinc oxide, the reduction to metallic zinc can lead to its migration to the surface and evaporation under sorption conditions [34]. On the other hand, the active components can form mixed oxides (spinels, perovskites, etc.) between each other or with a support. Mixed oxides are more resistant to the reduction and more active in H_2_S sorption [7,35,36].

The formation of mixed oxides is possible upon the sufficient calcination temperature being reached, otherwise the active phase will remain in the form of simple oxides [21,22]. It was found that the desulphurization decreases CuO, Cu_2_O > CuAl_2_O_4_, CuAlO_2_ > CuFe_2_O_4_, CuFeO_2_ >> Cu [10] with the condition that supports based on carbon materials cannot be used in oxidative regeneration.

In this paper, we aim to determine the effect of the chemical composition of the Zn-based sorbent on the ability of the material to absorb H_2_S. We present the results of the investigation of zinc-based sorbents produced by slurry impregnation of TiO_2_, SiO_2_ and Al_2_O_3_. We focus our analyses of Zn_2_TiO_4_ (ZT), ZnAl_2_O_4_ (ZA) and Zn_2_SiO_4_ (ZS) on the identification of the following physico-chemical properties of fresh materials: phase composition, susceptibility to reduction, pore structure and morphology. Subsequently, their sorption properties were tested in model gas desulphurization at 600 ${}^{\circ }$C with the regeneration at 650 ${}^{\circ }$C. The experimental results have been compared with the computational study of these materials, with respect to the ease of the incorporation of the sulphur in the surface and bulk layer of the respective oxides.

## 2. Experimental

### 2.1. Sorbents Preparation

Sorbents were prepared by the slurry impregnation method using the following reagents: zinc nitrate hexahydrate (≥99% Avantor), titanium dioxide (≥99% Panareac), aluminum oxide (>98.5% Avantor), hydrated silica (SiO_2_ x nH_2_O), natural clay (Drużkowska). TiO_2_, SiO_2_ and Al_2_O_3_ were used as the supports. The molar ratio of the precursors in the synthesis corresponded to that contained in the theoretically assumed for ZT, ZA and ZS mixed oxides. Weighed sample of the support was suspended in a solution of zinc nitrate under vigorous stirring for 24 h, then water evaporated (90 ${}^{\circ }$C) under stirring until the slurry was obtained. The obtained mass was dried and calcined at 900 ${}^{\circ }$C for 6 h. Subsequently, the material was crushed and mixed with a binder (Drużkowska clay) in 4:1 ratio. Then, it was formed in a matrix and dried, first in the air at room temperature and then in the oven. Finally, the sorbents were calcined for 6 h at 900 ${}^{\circ }$C and crushed to 0.4–0.8 mm. The details of sorbents preparation procedure have been described in our earlier paper [35].

### 2.2. Performance Tests

Tests of the desulphurization activity and regeneration of the sorbent were carried out in a tubular quartz reactor (d = 8 mm, L = 80 cm) placed in the three-zone furnace. A total of 1.5 cm${}^{3}$ of fresh sorbent (0.4–0.8 mm) was placed on an inert alumina. Model coal gas (H_2_—25%, CO—49.5%, CO_2_—20%, H_2_S—0.5%, CH_4_—5%vol.) was used for the tests of desulphurization activity. Mixture of 3% O_2_ vol. in N_2_ was used for sorbent regeneration. The bed was heated to 600 ${}^{\circ }$C and stabilized for 30 min in a nitrogen stream at the desulphurization test temperature. The content of hydrogen sulfide in the outlet gas from the reactor was determined by gas chromatograph (PerkinElmer Clarus 500) with FPD detector. Concentration of 100 ppm of H_2_S in the gas leaving reactor was considered as the moment of bed breakthrough. Then, the reactor was flushed with nitrogen and regeneration procedure was initiated. Regeneration started at 600 ${}^{\circ }$C in oxidizing atmosphere with temperature rise of 5 ${}^{\circ }$C/min to 650 ${}^{\circ }$C. This temperature was maintained in the reactor for a longer recovery time. The content of the sulphur oxide in the gaseous products of regeneration was determined using gas analyzer Mulator 610 (Maihak).

Sorption capacity was calculated using following formula:

(1) $$C_{S}=\frac{m_{Sads}}{m_{sorb}}=\frac{\left[\frac{P{}_{H_{2}S}^{ads}}{P{}_{H_{2}S}^{com}}\cdot \sum_{t}^{t_{0}}n_{in}\right]\cdot M_{S}}{m_{sorb}}=\frac{\left[\frac{\int_{0}^{t_{0}}\left(c{}_{H_{2}S}^{in}-c{}_{H_{2}S}^{out}\right)dt}{\int_{0}^{t_{0}}c{}_{H_{2}S}^{in}dt}\cdot \sum_{t}^{t_{0}}n_{H_{2}S}^{in}\right]\cdot M_{S}}{m_{sorb}}$$

where: $C_{S}$—mass of sulphur adsorbed by 1 gram of sorbent (g S/g); $m_{Sads}$—mass of sulphur adsorbed through the bed (g); $m_{sorb}$—sorbent mass (g); $P{}_{H_{2}S}^{ads}$—surface area of the adsorbed hydrogen sulfide (ppmv*h); $P{}_{H_{2}S}^{com}$—surface area of introduced hydrogen sulfide (ppmv*h); $n_{in}$—the number of moles of hydrogen sulfide introduced to the reactor until the breakthrough of the bed (mol); $c{}_{H_{2}S}^{in}$—the concentration of hydrogen sulfide fed to the reactor (ppmv); $c{}_{H_{2}S}^{out}$—the concentration of the hydrogen sulfide leaving the reactor (ppmv); $M_{S}$—molar mass of sulphur (g/mol).

### 2.3. Characterization

Reducibility of fresh sorbents was determined by the temperature-programmed reduction with hydrogen (TPRH2) in Pulse Chemisorb 2705’s Micromeritics apparatus with TCD detector. A total of 10 mg of the sample (particle size <0.08 mm) was placed in a reactor with a U-shaped tube and heated (5 ${}^{\circ }$C/min.) up to 900 ${}^{\circ }$C in the flow of 30 mL/min. of the mixture of 5% H_2_ vol. in argon.

The phase composition of materials was determined by X-ray powder diffraction (XRD), using X’Pert Pro diffractometer (PANalytical Ltd., Malvern, UK). The analyses of the samples of fresh, regenerated and reduced by hydrogen have been carried out. The diffractometer was equipped with the Ni filter and emitted CuKa radiation (λ = 1.5406 Å), the reflection was in the range of 2θ = 10–90. Crystallographic structure was determined on the basis of the recorded (using the program X’Pert HighScore Plus) and standard diffraction JCPDS. The average crystallite size of the dominant phase was determined from broadening of diffraction lines on the basis of the Scherrer equation.

The mercury porosimetry measurements were carried out using 440 Pascal CE Instruments, to the pressure of 200 MPa, on samples that were outgassed at room temperature for 1 h. The pore radius was calculated from the Washburn equation; calculation of the specific surface area based on a database—the granulometric patterns of pores.

### 2.4. Model and Computational Details

All calculations have been carried out using the DFT method with the VASP code version 5.4.1 [37,38,39,40,41]. The generalized gradient approximated PBE exchange-correlation functional has been employed [42] in line with other papers concerning the DFT simulations of related systems [43,44,45,46]. The choice of the pure PBE approximation can be justified by the fact it does not depend on arbitrary parameters such as Hubbard corrections. Although those have been reported to improve the band-gap characteristics of the Zn-based materials, fine tuning the parameters to fit the experimental data is beyond the scope of the present work, and it is unlikely to significantly affect the ground state energetics of the investigated systems. Therefore, we believe that the physical effects responsible for the binding energies, which are crucial from the point of view of the aim of this work, are represented correctly.

Projected Augmented Wave (PAW) method has been used, with the 500 eV energy cutoff to ensure the convergence with respect to the basis set. The Brillouin zone sampling was limited to 2 × 2 × 1 k-point mesh.

The structures have been relaxed to the forces smaller than 0.01 eV/Å. For the Zero Point Energy (ZPE) contribution, the finite displacement method was used with all atoms displaced by 0.0015 Å in order to estimate numerical Hessian matrix. All energies discussed in the manuscript have the meaning of Helmholtz free energies in 0 K, as they take into account the ZPE contribution to the internal energy of the system. They are marked as $E_{ZPE}$ (or $\Delta E_{ZPE}$ for energy differences).

The models represent the crystal lattice plane with (110) Miller indices of the spinel structure with the formula AB_2_O_4_. The structure is characterized by close-packed arrangement of the oxygen anions and two different types of metal sites: tetrahedric and octahedric. In a normal spinel structure (represented here by ZA sorbent), A (Zn) and B (Al) cations fill 1/8 of the tetrahedric and 1/2 of octahedric sites, respectively. In the inverse spinels (represented by ZT and ZS sorbents), the 1/2 of B-cations occupy tetrahedric sites as well, while all A-cations occupy octahedric sites. Other than that, the structures share the same geometry/symmetry [47].

**Figure 1:**
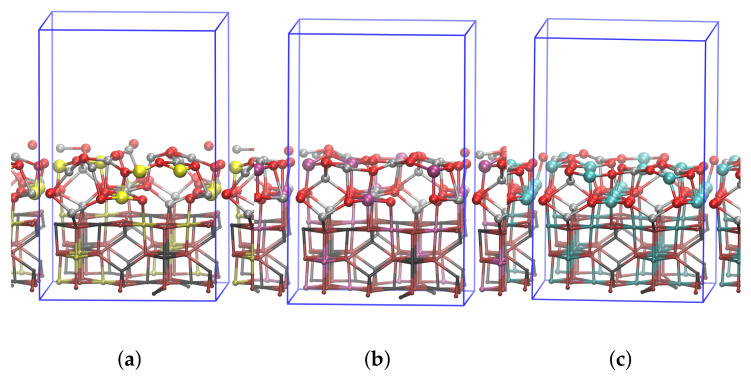
(**a**–**c**) lSpinel structures of the models used in simulations. Silicon, Titanium, Aluminum, Zinc and Oxygen are depicted in yellow, magenta, blue, gray and red, respectively. Three bottom layers that have been frozen in the simulations are shown in darker shade and as cylinders. Top two layers that were allowed to relax are shown in lighter shade in CPK representation. Blue frames represent the periodic box boundaries.

The vacuum slab of approximately 10 Å has been added on top of the spinel to simulate the surface. Three bottom layers have been held rigid during the simulation in order to account for bulk. Final lengths and angles of the simulation cell were a = b = 11.965 Å, c = 18.373 Å and α = β = γ = 90${}^{\circ }$. The geometries are shown in Figure 1.

Molecular structures have been plotted with VMD 1.9.3 [48].

## 3. Results and Discussion

### 3.1. Characterization of the Materials

#### 3.1.1. XRD Patterns

**Figure 2:**
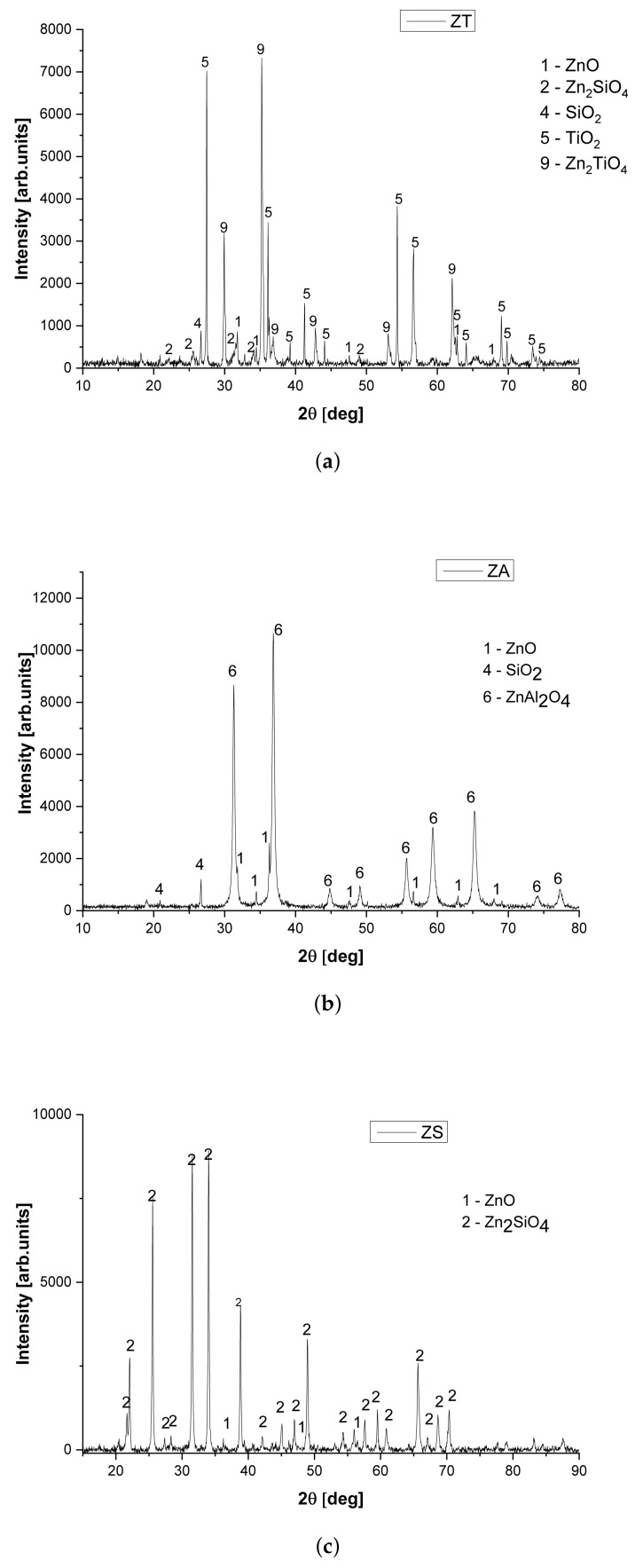
XRD pattern of fresh ZT sorbent (**a**), ZA sorbent (**b**) and ZS sorbent (**c**).

The X-ray diffraction patterns of zinc sorbents deposited on studied carriers are presented in Figure 2a–c. In the case of the ZT sorbent (Figure 2a), the dominant phase is zinc titanate (Zn_2_TiO_4_; JCPDS 00-025-1164), intense signals of TiO_2_ (JCPDS 00-021-1276, rutile) and ZnO (JCPDS 00-036-1451), SiO_2_ (JCPDS 00-033-1161) and Zn_2_SiO_4_ (JCPDS 00-037-1485) are also visible.

Peaks of ZnO are also detected on the diffraction pattern of the ZA sorbent (Figure 2b), as well as of the ZS (Figure 2c) in which dominant phase is the spinel Zn_2_SiO_4_ (JCPDS 00-037-1485). For the former, the dominant phase is zinc aluminate (JCPDS 00-005-0669). We conclude that the synthesis method used in this study does not ensure complete conversion of zinc oxide into mixed oxides, regardless of the support used.

Contrary to that, the zinc oxide deposited on the investigated carriers forms mixed metal oxides at 900 ${}^{\circ }$C, although weak signals of ZnO are still present, which indicates incomplete conversion. The average crystallite size of a dominant phase of studied materials (Table 1) ranged from 22.7 nm (ZA) to 51.2 nm (ZT). The largest and the smallest crystallites have been observed for the sorbents supported on TiO_2_ and on Al_2_O_3_, respectively. After the calcination of ZT at 900 ${}^{\circ }$C, the dominant phase is Zn_2_TiO_4_, which is consistent with the results of Meng [7]. The same temperature is sufficient to form Zn_2_SiO_4_ (ZS) and ZnAl_2_O_4_ (ZA).

The formation of the mixed oxides in the conditions assumed in this work is consistent with the literature reports, where the reader is referred to for a more detailed description. The calcination at 350 ${}^{\circ }$C was not sufficient to form a mixed metal oxide from ZnO and SiO_2_, as observed by Dhage et al. [22]. Similarly, the simple oxides ZnO and Al_2_O_3_ are still present after calcination at 400 ${}^{\circ }$C according to Tajizadegan et al. [21]. In addition, Wang et al. found that the formation of Zn_2_SiO_4_ needs the calcination of simple oxides for at least 1 h at 900 ${}^{\circ }$C [23].

#### 3.1.2. Morphology

Regardless of the support used, zinc oxide formed spinel-type mixed oxides. The specific surface area and the porosity of the material, as measured by mercury porosimetry (see Section 2.3), varied in the broad range. Table 1 gathers the values of the porosity and specific surface area for the investigated materials. The ZT and ZA sorbents showed the lowest specific surface area (SSA)—only 4.6 and 5.7 m${}^{2}$/g, respectively. On the other hand, the ZS sorbent showed significantly higher SSA—32.2 m${}^{2}$/g. However, the sorbent supported on silica (ZS) was characterized by the smallest average pore size—only 13.7 nm—while the ZT and ZA showed the average pore size of 310 and 541 nm, respectively. In general, the sorbents are characterized by small specific surface area and total porosity, which results from high calcination temperature.

In addition, ZA sorbent is characterized by the smallest average crystallite size—approximately 20 nm. The ZS and ZT sorbents contain crystallites approximately twice the size—45–50 nm; however, for the ZT sorbent, the size of the crystallites decreases to 31 nm after reduction at 900 ${}^{\circ }C$.

### 3.2. Reduction with H_2_

**Figure 3:**
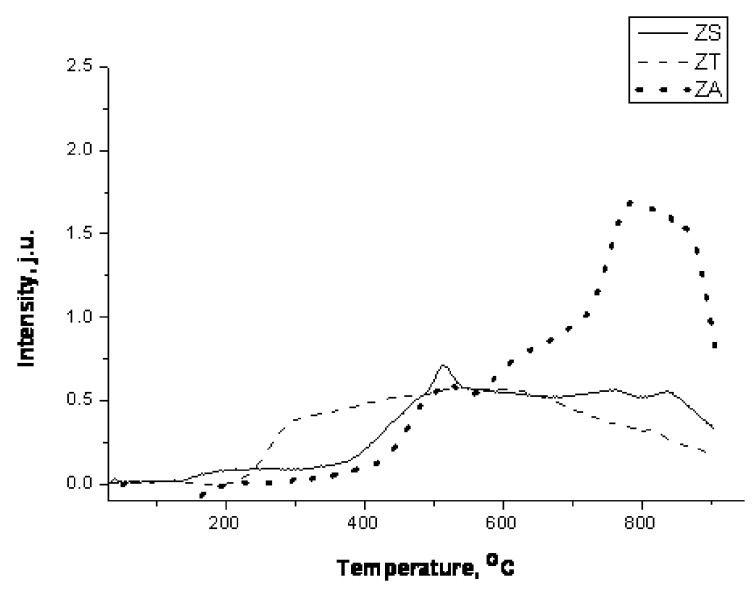
$TPRH_{2}$ profiles of ZT, ZS and ZA sorbents.

As the H_2_S sorption is the chemical process, the reactivity of the investigated sorbents is expected to play a significant role. Especially important seems to be the red-ox reactions (reactions (2)–(5)). Therefore, we have carried out the temperature-programmed reduction tests, with the H_2_ and the reducing agent. The results are summarized in Figure 3, which shows the $TPRH_{2}$ profiles of the investigated sorbents.

ZT shows a broad, relatively weak signal of hydrogen consumption from approximately 250 to 900 ${}^{\circ }$C, which is consistent with the literature reports [49], the presence of the ZnO phase in the material before the reduction, which shows broad and weak signals on $TPRH_{2}$ profiles [49].

In the reduced ZT sample, the dominant phase is Zn_2_Ti_3_O_8_ (JCPDS 00-038-0500). Moreover, there are strong signals coming from TiO_2_, ZnTiO_3_ (JCPDS 00-025-0671) and SiO_2_. The mixed oxides identified in the sorbent are formed by ZnO and TiO_2_; however, no ZnO has been identified. As it was shown by Hou [50], high temperature reduction of Zn_2_TiO_4_ in H_2_ atmosphere can decompose the mixed metal oxides into simple oxides: ZnO and TiO_2_. Alternatively, a spinel can undergo another transformation, such as partial reduction to ZnTiO_3_. This observation supports the broad peak observed in the $TPRH_{2}$ profile (see Figure 3).

Reduction of ZA starts at approximately 380 ${}^{\circ }$C and two peaks of H_2_ consumption are visible on the $TPRH_{2}$ profile: the first one shows maximum at 500 ${}^{\circ }$C, while the second one at 790 ${}^{\circ }$C. The only phase present in the material after the reduction is ghanite (ZnAl_2_O_4_). This is confirmed by the literature reports, in which the reduction of ZnAl_2_O_4_ to metallic Zn was reported to take place with use of carbon (graphite) [51]. Additionally, computer modeling indicates that during the reduction, with the increase in the temperature, ZnAl_2_O_4_ is partially degraded to simple oxides and then ZnO reacts with the reducing agent to form a metal phase [51]. Full reduction of ghanite was achieved at 1200 ${}^{\circ }$C, while intensive decomposition of ZnAl_2_O_4_ takes place above 900 ${}^{\circ }$C.

The ZS sorbent reacts with hydrogen at approximately 400 ${}^{\circ }$C and the temperature of maximum of H_2_ consumption is approximately 500 ${}^{\circ }$C. Similarly to the case of ZT sorbent, the peak is broad, which suggests the presence of the ZnO phase, which did not react with the support during the preparation of the sorbent. This phase undergoes the gradual reduction, resulting in one broad peak. In the reduced ZS sorbent, a dominant phase is Zn_2_SiO_4_ (JCPDS 00-008-0492), which changes the lattice parameters (JCPDS 00-037-1485) after reduction in the high temperature range.

**Figure 4:**
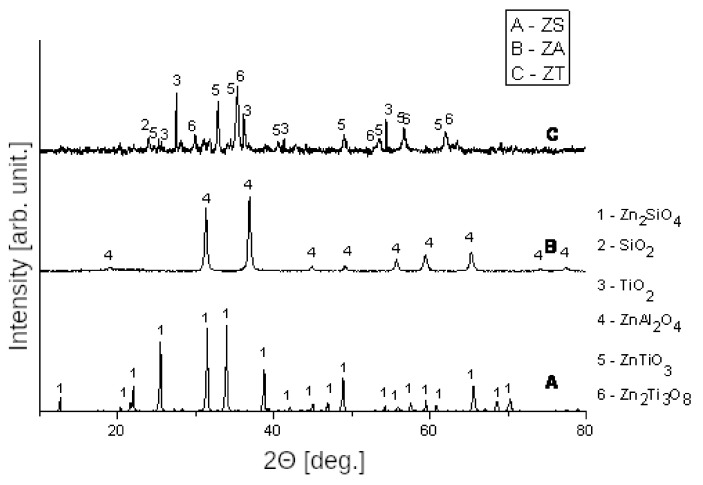
XRD spectra of sorbents ZT, ZS and ZA after reduction with hydrogen to a temperature of 900 ${}^{\circ }$C.

Interestingly, the metallic Zn cannot be observed in the XRD patterns of the reduced materials (see Figure 4), which is consistent with the evaporation of this form of the Zn in elevated temperatures. The possibility of the evaporation was described by Swisher et al. [34] to occur at 550–600 ${}^{\circ }C$, which is close to the temperature ranges in this work. That unfortunately means that the materials containing ZnO phase are susceptible to the Zn loss during the reduction. On the other hand, the preparation of mixed oxides with SiO_2_ prevents the reduction of ZnO by hydrogen. The sorbents prepared with the use of titanium dioxide as the carrier are the least resistant to the reduction in the hydrogen atmosphere.

### 3.3. Sulphur Absorption

The results of the model hot coal gas desulphurization (at 600 ${}^{\circ }$C) on the investigated sorbents are collected in Table 2. The largest amount of H_2_S was adsorbed during the first cycle by ZT sorbent. In subsequent cycles, the amount of H_2_S retained by this material increased almost twice compared to the first cycle. The degree of H_2_S removal from the gas by this sorbent was 99.7% in each cycle. The ZA sorbent shows the opposite properties—the amount of retained H_2_S decreases in subsequent cycles from 2.2 gS/100 g (first cycle) to 0.9 gS/100 g (third cycle). In the case of ZS, the amount of adsorbed H_2_S increases with successive work cycles, but during the first cycle it adsorbed only 0.7 gS/100 g, while during the third one—2.2 gS/100 g. The degree of gas purification from H_2_S by ZS increased with successive operation cycles from 98.0 to 99.6%.

The sorption capacity of the investigated materials increased in the order ZA < ZS < ZT and did not depend on their specific surface area, porosity or average crystallite size of the dominant phase. Instead, it could be correlated to the increasing reducibility of these materials observed in the $TPRH_{2}$ measurements. The reaction of ZnO with either H_2_S or H_2_ requires a cleavage of a metal–oxygen bond. The reaction of the metal oxide with H_2_S is endothermic, as is the reduction by hydrogen [7,52]. If ZnO is more stable in the structures of mixed oxides (spinels with TiO_2_, Al_2_O_3_ and SiO_2_), it is less reactive with respect to the hydrogen and more energy needs to be provided to allow for the reaction with H_2_S.

Therefore, it should be considered that susceptibility to reduction, which is associated with the phase composition of the sorbents, is one of the main parameters determining their sorption ability. This is evidenced by the results obtained by Tajizadegan—zinc oxide supported on Al_2_O_3_ (20% wt.) adsorbed 5.2 gS/100 g sorbent [21], which corresponds to a conversion degree of 64%. Commercial sorbent Sud-Chemie (G-72E) based on ZnO (90% wt.) adsorbed 3.2 gS/100 g, which results in a 9% degree of conversion. The investigated sorbents adsorb less hydrogen sulphide, especially in the first working cycle, which can be attributed to the difference in reactivity of both forms of ZnO: the simple oxide and the mixed oxide of spinel structure.

These considerations are confirmed by the results of XRD analysis of the sorbents after $TPRH_{2}$ tests. Zn_2_TiO_4_ is the only mixed oxide containing Zn that reacted with hydrogen. ZnAl_2_O_4_ appeared to be resistant to reduction, while Zn_2_SiO_4_ spinel recrystallized to another structure with a different unit cell.

### 3.4. Process Mechanism

From the atomistic point of view, the ability to incorporate the sulphur in the structure of the oxide can vary depending on stability of the sulphur atoms in the crystalline framework, accessibility of the sites and kinetics of the process. In this work, we have decided to focus on the first two effects and to neglect the latter. This approach was dictated by the high temperature of the process ranging up to 600 ${}^{\circ }$C, which allows to assume that activation barriers can be easily achieved, and the process is controlled thermodynamically rather than kinetically. This is consistent with the observations of Baird et al. [53], who found that kinetics of the process significantly decreases below 350 ${}^{\circ }$C. As the specific surface area of the materials has been obtained from the experiment and discussed above, the only factor that is assumed to play a role is thermodynamic stability of the particular sulphur sites.

Given the computational models used in the study, there are few particular effects that might influence the stability. First is the layer of atoms in the structure in which sulphur might be incorporated; second is the type of the site—tetrahedric or octahedric; and the last is the type of atoms surrounding this particular site—A or B cations. All these effects have been evaluated by means of the calculation of ΔG of the reaction, with the reference being the following assumed reactions:

(2) $$A_{n}B_{2n}O_{4n}+H2S⟶A_{n}B_{2n}O_{4n-1}OHSH$$

(3) $$A_{n}B_{2n}O_{4n-1}OHSH⟶A_{n}B_{2n}O_{4n-1}□S+H_{2}O$$

(4) $$A_{n}B_{2n}O_{4n-1}□S⟶A_{n}B_{2n}O_{4n-1}S$$

totaling to:

(5) $$A_{n}B_{2n}O_{4n}+H_{2}S⟶A_{n}B_{2n}O_{4n-1}S+H_{2}O$$

□ in Equations (3) and (4) represents the oxygen vacancy in the lattice. First step is the dissociative adsorption of H_2_S at the surface of the material (reaction (2)), giving rise to the surface -SH and -OH species. Upon the recombination (reaction (3)), the H_2_O molecule is formed and released, resulting in the vacancy in the lattice. Final step is the migration of the S atom to fill the vacancy. The vacancy can also migrate to the deeper layers, making not only top layer available for the sulphur incorporation. Due to very high number of particular elementary steps and in line with other works [46], only the ΔG of the total reaction has been considered as the most important criterion.

**Figure 5:**
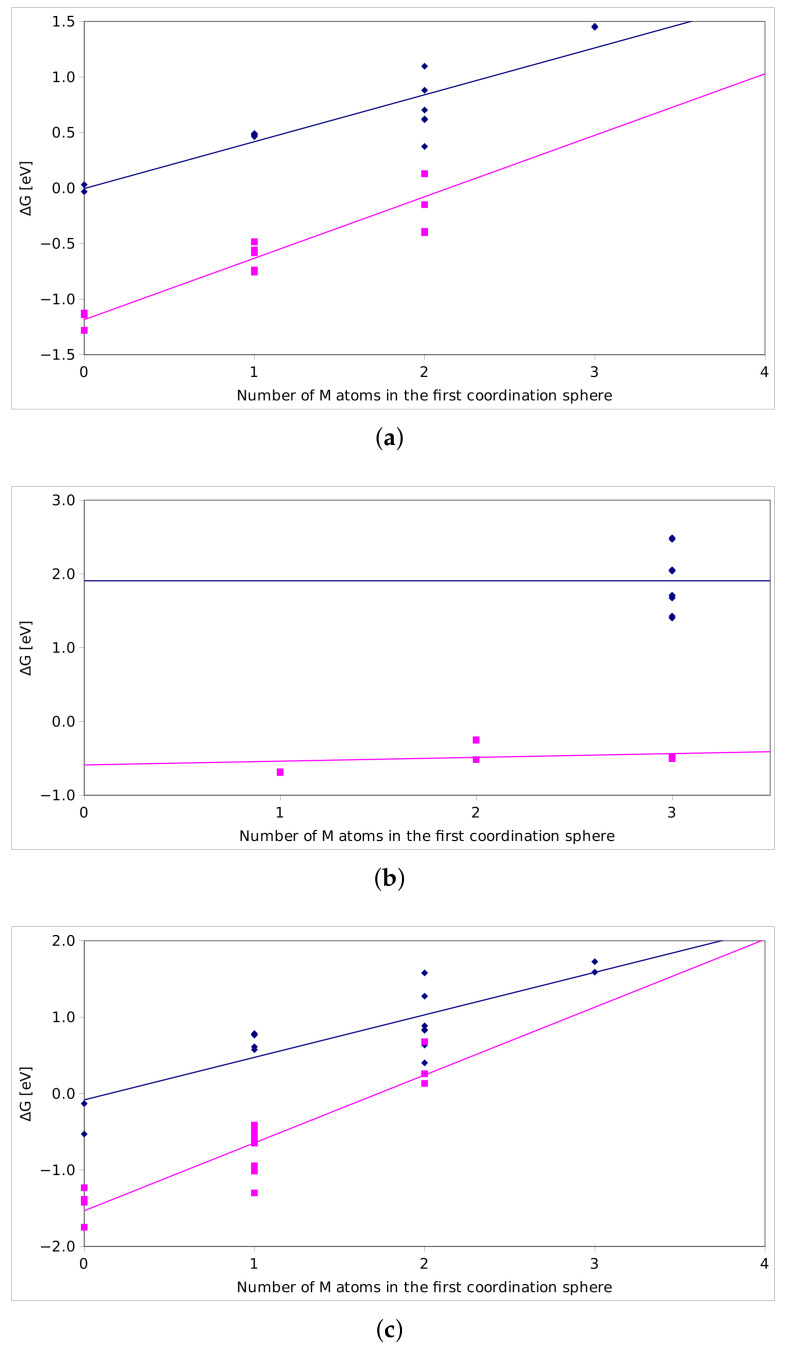
The relation between the ΔG and the number of Ti atoms (**a**), Al atoms (**b**) and Si atoms (**c**) in the first coordination sphere. The purple and blue series represent the location of the site in first and second atomic layer, respectively.

Figure 5 shows the ΔG dependence on the number of M atoms in the first coordination sphere. Here, the first coordination sphere is arbitrarily defined at 3.0 Å, but this value was selected to fit in between the cations in the investigated materials. It still needs to be clarified that, due to significant distortions of the crystal structure, the coordination spheres were not always clear.

We have observed similarities between the ZS and ZT systems, such as the greater ease of incorporation of S atom in the first layer than in the second one. However, for both ZS and ZT systems, there is significant dependence on the type of the neighbor in the first coordination shell. Figure 5a,c shows the positive slope of the ΔG as well for the first as the second layer substitutions.

There is a difference in the slope representing the first layer in the ZS system, suggesting that higher content of silicon is not promoting the incorporation of sulphur—for three silicon atoms in the first coordination sphere, the ΔG is positive. On the other hand, ZS system is the only one where the incorporation of S in the second layer is significantly more stable compared to the oxide—ΔG amounts to 0.5 eV for the most preferable case. ZT system behaves similarly to ZS with respect to dependence on the types of atoms in the first coordination sphere; however, the slope for the first layer of atoms is smaller. The ΔG for the most preferred substitution site is −1.3 eV compared to −1.8 eV for ZS system. On the other hand, even for two neighboring Ti atoms, the ΔG is negative, whereas for the ZS system it becomes positive for the relevant site with two Si atoms.

In the case of the ZA system, there is small to no influence of the binding of sulphur to the particular atoms, and the only role is played by the location of the site in the first or second layer. While it is thermodynamically preferable to incorporate the sulphur in the first layer, the energy gain is relatively small and amounts to 0.5 eV, on average. In addition, the second layer of atoms is not accessible energetically for the sulphur. It is possible that the structure reconstruction occurring in the experiment conditions will trap S atoms in the structure, but this is not a thermodynamically stable system. These results can explain a small amount of S absorbed by the material observed in the experiment.

It is important to note that there is no preference observed for the different neighbors in the first coordination shell for the ZA systems. In the second layer, all of the S sites occupy the site with three Al and one Zn atom; therefore, there is not enough different distributions. On the contrary, in the first layer, there are three different types of neighbors; however, the ΔG seems not to depend on the substitution at all.

The values of the ΔG for the sites of the different stability in the top and bulk layers are gathered in Table 3. We conclude that, for each of the investigated sorbents, the incorporation of the sulphur is preferred in the top layer. In addition, for ZT and ZS sorbents, the bulk layer is also able to provide partial stability of the sulphur. Contrary to that, the ZA sorbent is characterized by strong destabilization of the sites in the bulk layer upon the incorporation of sulphur. This is consistent with the experimental observation that, only in the first cycle, the sorbent is capable of absorbing the largest amount of the sulphur—which incorporates mostly in the top, most accessible layer.

## 4. Conclusions

We have investigated three different sorbents for the removal of the sulphur from the hot coal gas—ZT, ZA and ZS. The XRD analysis confirmed the most dominant phases in all three cases was the spinel-like Zn_2_TiO_4_, ZnAl_2_O_4_ and Zn_2_SiO_4_; however, a significant amount of ZnO was also present. We have determined that the specific surface area and the pore volume do not play an important role in the sulphur removal process. Instead, the most important parameter is the reducibility of the material, which correlates to the TPRH2 curves.

The best properties among the investigated systems have been exhibited by the ZT sorbent, in which the sorption capacity reaches over 25 g/100 g and increases in the consecutive work cycles. Contrary to that, the sorption ability of the ZA material decreases, which corresponds well to the computer simulation results, showing the unfavorable thermodynamics of the migration of the S atoms to the bulk. ZS sorbent is also characterized by an increase in the efficiency along the work cycle; however, the amount of sulphur captured is smaller than for the ZT.

## Figures and Tables

**Table 1:** The specific surface area, total porosity and the average crystallite size of the dominant phase for sorbents: ZT, ZA, ZS.

Parameter—Sorbent	ZT	ZA	ZS
Total pore volume (cm${}^{3}$/g)	0.19	0.23	0.38
Specific surface area (m${}^{2}$/g)	4.6	5.7	32.2
Average pore size (nm)	310	541	13.7
Porosity (%)	45.9	41.9	48.5
Average crystallite size of the dominant phase (fresh sorbent) (nm)	51.2 (Zn_2_TiO_4_)	22.7 (ZnAl_2_O_4_)	45.0 (Zn_2_SiO_4_)
Average crystallite size after reduction at 900 ${}^{\circ }$C (nm)	31.6 (Zn_2_TiO_4_)	25.5 (ZnAl_2_O_4_)	46.1 (Zn_2_SiO_4_)

**Table 2:** Results of the H_2_S sorption from model gas at 600 ${}^{\circ }$C.

Sorbent	Work Cycle	gS/100 g	% Capacity	% Purification
ZT	1 2 3	2.3 3.8 4.2	14.8 23.2 25.6	99.7 99.7 99.7
ZA	1 2 3	2.2 0.9 0.8	15.9 6.3 5.7	97.2 99.0 99.0
ZS	1 2 3	0.7 1.2 2.2	5.8 10.7 22.7	98.0 99.1 99.6

**Table 3:** The ΔG of the most stable, least stable and their average for the investigated sorbents.

Sorbent	Most Stable Site (eV)	Least Stable Site (eV)	Average (eV)
ZT top layer	−1.3	0.1	−0.8
ZT bulk layer	0.0	1.5	0.6
ZA top layer	−0.7	−0.3	−0.5
ZA bulk layer	1.4	2.5	1.9
ZS top layer	−1.7	0.7	−0.7
ZS bulk layer	−0.5	1.7	0.8

## Data Availability

The data is available on reasonable request from the corresponding author.
